# Supplementary material for: A Dietary Intervention of Bioactive Enriched Foods Aimed at Adults at Risk of Metabolic Syndrome: Protocol and Results from PATHWAY-27 Pilot Study
Source: Nutrients. 2019 Aug 6;11(8):1814. doi: 10.3390/nu11081814 (PMC6723599; doi:10.3390/nu11081814)
Supplement: Supplementary file 1 [file nutrients-11-01814-s001.pdf]

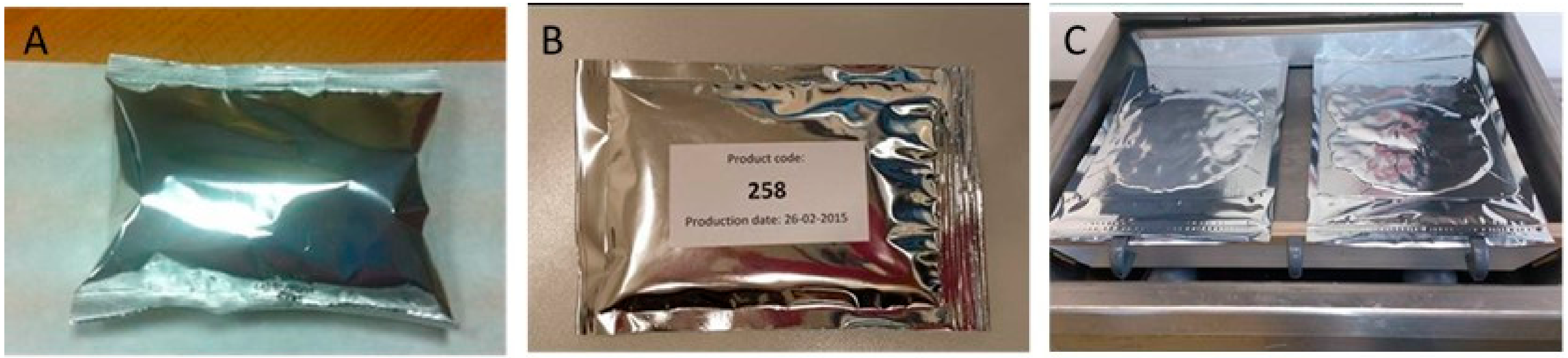

Figure S1. Packaging used in PATHWAY-27. (**A**) biscuits, (**B)** milkshake powder, (**C**) pancakes.

Table S1. Quantitative analysis of SCFA and MCFA in fasting serum at T0 and T28 of the dairy intervention.

	**Concentration in fasting serum (μM)**															
	**acetic acid**		**propionic acid**		**isobutyric acid**		**butyric acid**		**2-methylbutyric + isovaleric acid**		**valeric acid**		**Hexanoic acid**		**Heptanoic acid**	
	**T0**	**T28**	**T0**	**T28**	**T0**	**T28**	**T0**	**T28**	**T0**	**T28**	**T0**	**T28**	**T0**	**T28**	**T0**	**T28**
**AC**	22.86	47.81	4.09	4.29	2.65	2.24	2.315	2.675	1.674	4.099	0.148	0.175	0.661	0.73	0.132	0.168
**AC**	79.82	39.27	4.17	3.66	1.81	1.94	3.056	2.261	2.713	2.42	0.291	0.16	1.046	0.736	0.202	0.117
**AC**	26.16	35.95	2.79	3.69	2.2	1.98	1.68	2.625	1.135	2.048	0.164	0.227	0.708	0.785	0.129	0.158
**AC**	21.35	24.1	3.74	3.51	2.03	2.53	2.153	2.066	0.888	1.026	0.646	0.68	0.794	0.644	0.152	0.138
**AC**	23.43	29.61	3.09	3.28	2.01	2.14	1.921	1.703	1.737	3.383	0.144	0.161	0.734	0.702	0.181	0.134
**AC**	44.73	40.52	7.81	5.73	3.63	3.1	8.274	4.248	2.476	2.769	0.752	0.668	0.473	0.516	0.101	0.112
**AC**	26.92	1041.32*	2.74	3.3	1.9	2.21	1.817	2.568	1.237	1.934	0.117	0.134	0.701	1.061	0.156	0.423
**AC**	25.76	231.65	2.94	3.13	1.73	1.84	1.646	1.786	1.389	2.002	0.124	0.172	0.52	0.698	0.088	0.11
**AC**	18.32	20.16	2.33	2.2	1.89	1.71	1.158	1.051	1.215	1.666	0.112	0.134	0.557	0.587	0.097	0.116
**AC**	17.51	23.02	2.21	2.37	1.98	1.74	1.281	1.467	1.024	2.146	0.098	0.138	0.41	0.502	0.095	0.105
**AC**	23.87	25.57	3.57	4.4	1.96	2.36	2.108	1.769	1.645	1.957	0.144	0.126	0.596	0.569	0.121	0.101
**AC**	18.53	17.66	2.62	2.16	1.9	1.43	1.392	1.255	1.154	1.086	0.117	0.13	0.782	0.713	0.125	0.119
**AC**	21.17	26.62	2.44	2.17	1.66	1.62	1.262	1.214	2.13	1.619	0.094	0.096	0.558	0.679	0.114	0.14
**AC**	28.61	26.33	2.3	2.47	1.96	1.93	1.24	1.739	1.424	1.206	0.113	0.164	0.636	0.937	0.115	0.149

**AC**	27.54	41.65	2.97	4.08	2.04	1.9	1.934	3.463	2.183	2.845	0.182	0.309	0.672	0.847	0.12	0.13
**mean**	**28.439**	**44.994**	**3.321**	**3.363**	**2.090**	**2.045**	**2.216**	**2.126**	**1.602**	**2.147**	**0.216**	**0.232**	**0.657**	**0.714**	**0.129**	**0.148**
**OBG**	26.49	24.93	3.73	3.32	2.12	2.18	2.406	2.429	3.727	1.045	0.242	0.172	0.663	0.648	0.114	0.125
**OBG**	18.72	23.46	3.04	2.63	1.86	1.76	1.91	1.92	1.202	1.773	0.119	0.219	0.487	0.712	0.117	0.125
**OBG**	17.44	37.33	2.49	2.82	1.71	2.02	1.128	1.591	0.961	1.818	0.103	0.113	0.598	0.679	0.118	0.094
**OBG**	23.98	28.95	2.81	3.02	1.91	1.95	1.345	1.561	2.026	2.182	0.097	0.155	0.77	0.83	0.105	0.116
**OBG**	37.29	24.48	2.67	2.72	1.82	2.25	2.053	1.64	1.344	1.374	0.114	0.107	0.54	0.571	0.101	0.101
**OBG**	24.78	40.03	2.21	3.2	1.83	2.11	2.02	3.005	1.852	1.695	0.141	0.351	0.552	0.547	0.091	0.132
**OBG**	21	20.42	2.27	1.85	1.44	1.45	1.303	1.007	0.806	0.908	0.081	0.09	0.539	0.49	0.101	0.091
**OBG**	17.02	18.89	2.03	2.78	1.41	1.53	0.919	1.168	0.838	0.995	0.087	0.104	0.448	0.438	0.099	0.119
**OBG**	39.43	61.21	3.14	4.2	2.06	1.94	2.117	3.248	1.523	1.783	0.174	0.227	0.871	0.585	0.134	0.108
**OBG**	25.49	28.85	2.8	3.05	2.05	1.86	1.61	1.742	1.458	1.963	0.173	0.17	0.805	0.667	0.161	0.137
**OBG**	18.65	20.21	2.45	2.6	1.9	2.03	1.355	1.451	1.054	1.146	0.115	0.106	0.927	0.779	0.132	0.125
**OBG**	24.79	18.45	3.14	2.6	1.85	1.52	1.983	1.41	1.407	1.47	0.157	0.159	0.578	0.41	0.109	0.091
**mean**	**24.59**	**28.93**	**2.73**	**2.90**	**1.83**	**1.88**	**1.68**	**1.85**	**1.52**	**1.51**	**0.13**	**0.16**	**0.65**	**0.61**	**0.12**	**0.11**
**DHA**	23.56	48.74	2.88	3.2	1.68	1.79	2.226	2.121	3.198	1.578	0.12	0.094	0.635	0.79	0.138	0.123
**DHA**	39.9	39	3.11	2.87	1.95	1.87	1.87	1.429	1.797	1.961	0.126	0.09	0.695	0.534	0.158	0.114
**DHA**	38.35	30.79	3.66	3.81	2.28	2.81	2.262	2.171	2.531	2.654	0.133	0.143	1.2	1.251	0.131	0.13
**DHA**	38.91	40.17	3.06	2.86	2.09	2.05	1.558	1.517	1.706	2.643	0.104	0.11	0.684	0.712	0.109	0.112
**DHA**	18.32	27.6	2.52	2.74	1.47	1.74	1.404	1.472	0.991	1.11	0.234	0.126	0.833	0.677	0.281	0.133
**DHA**	34.49	30.37	3.05	2.84	1.61	1.76	1.654	1.42	1.045	2.064	0.103	0.112	0.492	0.477	0.157	0.121
**DHA**	57.42	40.71	3.01	3.37	1.88	1.64	2.982	3.821	1.609	1.622	0.213	0.283	0.581	0.653	0.117	0.215
**DHA**	30.77	52.91	2.83	3.36	1.82	2.11	1.775	2.715	2.181	2.87	0.118	0.155	0.637	0.714	0.124	0.107
**DHA**	22.49	44.24	2.25	3.36	1.74	1.89	1.13	2.02	1.141	2.218	n.d	0.134	0.492	0.541	0.088	0.09
**DHA**	56.26	62.3	3.14	2.68	2.26	2.26	2.044	1.432	2.347	1.6	0.139	0.134	0.535	0.638	0.114	0.115
**DHA**	22.79	29.35	2.96	4.46	1.67	2.4	1.549	2.072	1.433	2.471	0.182	0.229	0.775	0.691	0.207	0.142
**DHA**	35.14	42.22	2.54	2.25	1.84	1.85	1.696	1.632	1.215	1.257	0.102	0.158	0.601	0.712	0.115	0.115
**DHA**	50.22	26.25	2.92	2.32	1.84	1.83	1.542	1.336	1.888	3.742	0.117	0.152	0.532	0.525	0.105	0.112
**DHA**	27.63	19.11	2.59	2.53	1.78	1.72	1.791	1.63	1.701	1.371	0.127	0.109	0.597	0.645	0.134	0.16
**mean**	**35.45**	**38.13**	**2.89**	**3.05**	**1.85**	**1.98**	**1.82**	**1.91**	**1.77**	**2.08**	**0.14**	**0.14**	**0.66**	**0.68**	**0.14**	**0.13**
**DHA+AC**	24.79	28.61	3.22	3.29	1.59	1.56	1.821	1.712	0.763	1.197	0.152	0.161	1.042	0.604	0.176	0.113
**DHA+AC**	25.8	30.71	2.95	3.6	1.92	2.34	1.39	1.61	1.419	1.757	0.138	0.219	0.626	0.72	0.144	0.192
**DHA+AC**	21.73	39.77	3.19	3.45	2.26	1.95	1.662	2.181	2.446	2.836	0.133	0.156	0.699	0.7	0.137	0.171
**DHA+AC**	27.95	25.29	3.33	2.61	1.9	1.89	2.773	1.83	2.672	2.441	0.143	0.143	0.686	0.626	0.118	0.107
**DHA+AC**	32.92	32.08	3.64	3.03	2.09	1.85	1.972	1.886	1.961	2.004	0.198	0.154	0.96	0.689	0.205	0.14
**DHA+AC**	30.93	30.06	2.88	2.5	1.89	1.68	2.04	1.936	1.08	1.372	0.147	0.123	0.701	0.729	0.15	0.146

**DHA+AC**	29.22	54.02	3.34	6.39	2.16	2	2.212	4.325	2.838	2.298	0.259	0.262	0.824	0.704	0.189	0.12
**DHA+AC**	18.84	25.03	2.55	2.42	1.97	2.14	1.263	1.716	1.078	1.139	0.078	0.107	0.723	0.67	0.102	0.126
**DHA+AC**	23.25	25.98	2.93	2.38	1.67	1.42	2.377	1.669	1.954	1.293	0.61	0.404	0.64	0.581	0.151	0.111
**DHA+AC**	22.69	29.28	2.84	3.34	1.77	1.89	1.739	2.038	1.894	1.838	0.088	0.162	0.698	0.632	0.099	0.115
**DHA+AC**	23.88	20.37	2.52	2.34	1.79	1.8	1.291	1.338	1.455	1.121	0.105	0.127	0.682	0.558	0.098	0.088
**DHA+AC**	19.81	21.65	2.31	2.39	1.53	1.64	1.622	1.48	1.017	1.101	0.079	0.087	0.704	0.551	0.088	0.097
**mean**	**25.15**	**30.24**	**2.98**	**3.15**	**1.88**	**1.85**	**1.85**	**1.98**	**1.71**	**1.70**	**0.18**	**0.18**	**0.75**	**0.65**	**0.14**	**0.13**
**DHA+OB**	23.27	23.23	2.85	3.1	1.96	2.65	1.694	2.233	1.135	3.595	0.103	0.132	0.523	0.597	0.125	0.121
**DHA+OB**	18.09	29.67	2.86	3.63	1.85	2.28	1.613	2.302	1.797	4.149	0.205	0.187	0.883	0.749	0.239	0.165
**DHA+OB**	52.98	30.77	2.65	2.57	1.73	1.77	1.742	1.481	2.044	1.736	0.091	0.109	0.731	0.558	0.106	0.134
**DHA+OB**	21.22	31.78	4.05	4.4	1.8	1.98	2.121	2.339	1.341	1.686	0.239	0.277	0.56	0.522	0.143	0.12
**DHA+OB**	194.62	162.79	3	2.71	2.39	1.94	1.501	1.546	0.834	1.88	0.161	0.119	0.774	0.668	0.183	0.166
**DHA+OB**	22.21	31.26	2.17	2.45	1.46	1.59	1.169	1.391	1.271	1.77	0.14	0.097	1.101	0.862	0.149	0.1
**DHA+OB**	21.93	23.4	3.14	3.81	2.28	2	2.064	2.632	1.885	1.996	0.136	0.315	0.772	0.511	0.106	0.087
**DHA+OB**	23.73	30.12	3.05	3.11	2.08	2.09	1.818	2.226	1.584	1.676	0.112	0.098	0.639	0.597	0.094	0.102
**DHA+OB**	14.75	17.67	2.36	2.46	1.88	1.64	1.153	1.636	1.321	1.813	0.122	0.401	0.59	0.916	0.102	0.452
**DHA+OB**	26.29	39.6	2.39	2.28	1.62	1.83	1.33	1.359	1.289	2.332	0.137	0.104	0.904	0.77	0.193	0.117
**DHA+OB**	17.81	22.48	2.93	2.53	1.97	1.78	2.101	1.557	1.523	1.715	0.307	0.145	0.979	0.529	0.202	0.117
**DHA+OB**	17.33	28.26	2.37	2.43	1.67	1.95	1.37	1.82	1.167	1.753	0.108	0.173	0.565	0.538	0.124	0.109
**DHA+OB**	14.07	15.73	1.91	1.99	1.53	1.7	1.05	1.232	1.162	0.775	n.d	0.095	0.483	0.494	0.1	0.092
**mean**	**36.02**	**37.44**	**2.75**	**2.88**	**1.86**	**1.94**	**1.59**	**1.83**	**1.41**	**2.07**	**0.16**	**0.17**	**0.73**	**0.64**	**0.14**	**0.14**

* Value was removed as an outlier. Concentrations are given in µM. n.d – not detected.

Table S2. Quantification of main polyphenol metabolites in fasting serum at T0 and T28 of the dairy intervention.

	**Concentration in fasting serum (µM)**													
	**4-hydroxybenzoic acid**		**3-(3-hydroxyphenyl propanoic acid**		**vanillic acid**		**homovanillic acid**		**hippuric acid**		**t-ferulic acid**		**Cinnamic acid**	
	**T0**	**T28**	**T0**	**T28**	**T0**	**T28**	**T0**	**T28**	**T0**	**T28**	**T0**	**T28**	**T0**	**T28**
**AC**	n.d.	n.d.	0.44	0.74	0.02	0.81	0.13	0.13	1.97	6.04	n.d.	n.d.	9.74	9.36
**AC**	n.d.	n.d.	1.06	2.10	0.03	0.07	0.12	0.49	7.89	12.38	n.d.	n.d.	9.75	10.15
**AC**	n.d.	n.d.	0.90	1.09	0.01	0.01	0.09	0.14	24.15	15.81	n.d.	n.d.	9.44	9.15
**AC**	n.d.	n.d.	0.35	0.34	0.02	0.01	0.16	n.d.	3.20	1.46	n.d.	n.d.	9.30	6.98
**AC**	n.d.	n.d.	1.66	1.84	n.d.	0.03	0.29	0.14	7.61	12.57	n.d.	n.d.	8.69	8.52
**AC**	n.d.	n.d.	1.42	0.73	0.02	0.01	0.09	0.12	9.91	3.68	n.d.	0.00	8.94	9.62
**AC**	1.27	1.84	0.12	0.38	0.01	0.01	0.08	0.07	1.22	0.83	n.d.	n.d.	8.96	9.10
**AC**	n.d.	n.d.	0.43	0.57	0.01	0.00	0.15	0.17	5.14	11.28	n.d.	0.01	9.23	9.09

**AC**	n.d.	n.d.	0.44	0.96	n.d.	0.01	0.09	0.16	6.60	11.48	n.d.	n.d.	9.02	8.73
**AC**	n.d.	n.d.	0.69	1.04	0.00	0.01	0.09	0.12	7.66	5.03	n.d.	n.d.	9.07	8.52
**AC**	n.d.	n.d.	1.11	1.71	0.01	0.03	0.18	0.20	22.37	11.54	n.d.	n.d.	10.64	8.61
**AC**	n.d.	n.d.	2.65	0.80	0.01	0.02	0.12	0.27	11.43	6.80	n.d.	n.d.	8.15	8.91
**AC**	n.d.	n.d.	0.52	1.84	n.d.	0.01	0.10	0.13	18.63	16.18	n.d.	n.d.	9.37	9.16
**AC**	n.d.	n.d.	2.40	3.76	0.02	0.01	0.17	0.20	7.83	4.33	0.01	n.d.	8.62	9.08
**AC**	n.d.	n.d.	0.47	0.51	n.d.	0.01	0.05	n.d.	3.65	4.23	n.d.	n.d.	8.48	9.51
**mean**	**1.27**	**1.84**	**0.98**	**1.23**	**0.02**	**0.07**	**0.13**	**0.18**	**9.28**	**8.24**	**0.01**	**0.00**	**9.16**	**8.96**
**OBG**	n.d.	n.d.	1.02	1.08	0.00	0.01	0.04	0.09	21.12	8.55	n.d.	n.d.	7.46	8.58
**OBG**	n.d.	n.d.	1.11	0.82	0.01	0.02	0.04	0.18	4.23	5.50	n.d.	n.d.	8.52	9.88
**OBG**	n.d.	0.12	1.28	2.09	0.01	0.04	0.13	0.20	6.96	4.33	n.d.	n.d.	8.92	11.14
**OBG**	n.d.	n.d.	0.19	0.65	0.39	0.50	0.13	0.06	4.27	4.39	n.d.	n.d.	9.07	9.58
**OBG**	n.d.	n.d.	0.55	0.69	0.02	0.01	0.14	0.17	5.88	2.89	n.d.	n.d.	9.84	9.97
**OBG**	n.d.	n.d.	0.59	1.06	0.01	0.01	0.09	0.06	2.72	6.19	n.d.	n.d.	9.11	8.80
**OBG**	n.d.	n.d.	0.74	0.72	n.d.	0.06	0.10	0.18	15.84	7.22	n.d.	n.d.	9.48	9.78
**OBG**	n.d.	n.d.	0.66	1.26	n.d.	0.02	0.05	0.10	4.14	7.98	0.01	n.d.	8.42	8.52
**OBG**	n.d.	n.d.	1.27	1.20	0.01	0.01	0.10	0.16	2.92	2.33	n.d.	n.d.	9.97	8.67
**OBG**	n.d.	n.d.	1.07	1.02	0.03	0.01	0.18	0.07	13.54	8.35	0.01	n.d.	10.06	8.17
**OBG**	n.d.	n.d.	1.10	0.47	0.02	0.36	0.14	0.06	2.10	3.56	n.d.	n.d.	10.18	8.82
**OBG**	n.d.	n.d.	0.89	0.94	0.01	n.d.	0.06	0.24	11.89	5.87	n.d.	n.d.	9.65	9.69
**mean**	**n/a**	**0.12**	**0.87**	**1.00**	**0.05**	**0.09**	**0.10**	**0.13**	**7.97**	**5.60**	**0.01**	**n/a**	**9.22**	**9.30**
**DHA**	n.d.	n.d.	0.87	2.10	0.02	0.02	0.10	0.30	6.60	8.52	n.d.	n.d.	8.81	9.23
**DHA**	n.d.	n.d.	5.28	1.01	0.02	0.01	0.30	0.21	11.47	1.80	n.d.	n.d.	9.35	10.10
**DHA**	n.d.	n.d.	0.45	0.69	0.03	0.02	0.14	0.09	7.64	6.47	n.d.	n.d.	7.04	9.61
**DHA**	n.d.	0.65	0.71	0.52	n.d.	0.02	0.09	0.10	7.34	3.79	n.d.	n.d.	9.89	10.12
**DHA**	n.d.	n.d.	0.78	0.78	0.01	0.01	0.07	0.13	4.18	6.30	n.d.	n.d.	9.53	9.23
**DHA**	n.d.	n.d.	1.42	2.59	0.02	0.02	0.09	0.11	4.17	3.93	n.d.	n.d.	8.83	9.71
**DHA**	n.d.	n.d.	2.71	1.39	0.02	0.01	0.30	0.12	37.25	30.29	0.01	0.00	11.44	9.30
**DHA**	n.d.	n.d.	3.73	3.94	0.01	0.00	0.05	0.12	2.88	10.03	n.d.	n.d.	8.89	9.42
**DHA**	n.d.	n.d.	0.97	1.24	n.d.	0.01	0.07	0.24	6.16	9.93	n.d.	n.d.	7.95	9.71
**DHA**	n.d.	n.d.	2.15	1.13	0.02	0.03	0.42	0.12	8.57	5.30	n.d.	n.d.	9.24	7.96
**DHA**	n.d.	n.d.	1.10	1.73	0.01	0.01	0.05	0.21	10.89	10.27	n.d.	n.d.	8.98	9.98
**DHA**	n.d.	n.d.	0.50	0.35	0.01	0.01	0.11	0.17	9.08	5.36	n.d.	n.d.	9.03	9.25
**DHA**	n.d.	0.46	1.15	1.22	0.00	0.04	n.d.	0.10	6.77	13.97	n.d.	0.00	9.71	9.87
**DHA**	n.d.	n.d.	1.05	1.60	0.00	0.01	0.11	0.23	1.11	0.91	n.d.	n.d.	7.86	9.63
**mean**	**n/a**	**0.56**	**1.63**	**1.45**	**0.01**	**0.02**	**0.14**	**0.16**	**8.86**	**8.35**	**0.01**	**0.00**	**9.04**	**9.51**
**DHA+AC**	0.12	n.d.	0.71	0.70	0.01	0.01	0.06	0.08	9.89	9.11	n.d.	0.00	9.04	8.51
**DHA+AC**	n.d.	n.d.	0.24	0.52	0.02	0.01	0.17	0.15	5.00	2.34	n.d.	n.d.	10.13	8.92

**DHA+AC**	n.d.	n.d.	0.65	0.89	0.02	0.02	0.09	0.04	5.30	19.16	0.01	n.d.	9.67	10.01
**DHA+AC**	n.d.	n.d.	1.06	0.45	0.02	n.d.	0.09	0.09	3.17	3.00	n.d.	n.d.	8.59	8.86
**DHA+AC**	n.d.	n.d.	0.88	1.45	0.01	0.04	0.08	0.07	5.23	6.58	n.d.	n.d.	10.08	10.19
**DHA+AC**	n.d.	n.d.	1.37	0.97	0.05	0.02	0.17	0.14	2.16	4.69	n.d.	0.01	9.81	8.35
**DHA+AC**	n.d.	n.d.	0.96	1.06	0.01	0.01	0.15	0.08	4.76	5.62	n.d.	n.d.	8.40	8.56
**DHA+AC**	n.d.	n.d.	0.66	0.73	0.02	0.01	0.21	0.13	3.48	3.20	n.d.	n.d.	9.36	9.83
**DHA+AC**	n.d.	n.d.	2.26	1.60	0.01	0.02	0.16	0.08	6.66	8.81	0.01	n.d.	9.47	9.51
**DHA+AC**	n.d.	n.d.	0.44	1.30	n.d.	0.03	0.10	0.20	11.44	12.90	n.d.	n.d.	9.25	8.91
**DHA+AC**	n.d.	n.d.	0.41	0.75	0.01	0.01	0.09	n.d.	2.87	12.66	n.d.	n.d.	7.22	10.46
**DHA+AC**	n.d.	n.d.	0.51	0.25	0.01	0.01	0.16	0.08	8.64	4.01	n.d.	n.d.	9.94	8.29
**mean**	**0.12**	**n/a**	**0.85**	**0.89**	**0.02**	**0.02**	**0.12**	**0.10**	**5.72**	**7.67**	**0.01**	**0.00**	**9.25**	**9.20**
**DHA+OB**	n.d.	n.d.	0.66	1.06	n.d.	0.01	0.10	0.17	5.80	2.80	n.d.	n.d.	10.12	9.97
**DHA+OB**	n.d.	n.d.	0.33	0.34	0.02	0.03	0.09	0.06	3.56	1.57	n.d.	n.d.	10.04	10.02
**DHA+OB**	n.d.	n.d.	3.75	1.00	0.01	0.01	0.29	0.16	32.30	8.33	0.00	n.d.	11.09	7.94
**DHA+OB**	n.d.	n.d.	2.74	0.78	0.01	0.03	0.16	0.08	6.00	4.97	n.d.	n.d.	9.87	9.15
**DHA+OB**	n.d.	n.d.	0.71	1.03	0.02	0.02	0.16	0.19	3.51	6.70	n.d.	0.00	10.23	9.78
**DHA+OB**	n.d.	n.d.	1.15	1.17	0.01	0.01	0.13	0.08	7.99	10.48	n.d.	n.d.	10.19	10.43
**DHA+OB**	n.d.	n.d.	1.54	1.54	0.01	0.01	0.11	0.12	6.29	5.81	n.d.	n.d.	9.78	8.17
**DHA+OB**	n.d.	n.d.	1.72	1.54	0.01	0.02	0.31	0.18	14.88	10.78	n.d.	n.d.	8.59	9.58
**DHA+OB**	n.d.	n.d.	1.68	1.18	0.01	0.03	0.07	0.07	3.45	6.27	n.d.	n.d.	9.71	9.83
**DHA+OB**	n.d.	n.d.	1.44	0.85	n.d.	0.01	0.19	0.19	6.56	1.88	n.d.	n.d.	8.72	8.94
**DHA+OB**	n.d.	n.d.	0.26	0.53	0.00	0.01	0.19	0.14	9.90	8.26	n.d.	n.d.	9.11	8.80
**DHA+OB**	n.d.	n.d.	0.53	3.22	0.01	0.02	0.12	0.10	3.57	4.21	n.d.	n.d.	7.85	9.64
**DHA+OB**	n.d.	n.d.	1.77	3.31	n.d.	0.01	0.13	0.15	1.42	8.64	0.01	0.00	8.82	9.34
**mean**	**n/a**	**n/a**	**1.40**	**1.35**	**0.01**	**0.02**	**0.16**	**0.13**	**8.09**	**6.21**	**0.01**	**0.00**	**9.55**	**9.35**

Concentration is given in µM. Following compounds were found <LOD: 2,6-diOH-benzoic acid, 3,5-diOH-benzoic acid, 2,5-diOH-benzoic acid, protocatechuic acid, gallic acid, p-coumaric acid, m-coumaric acid, o-coumaric acid, 3-4hydroxyphenyl propionic acid, 3-hydroxyphenyl acetic acid, 3,4-dihydroxyphenyl acetic acid, alpha-hydroxyhippuric acid, caffeic acid, ellagic acid, sinapic acid, syringic acid, urolithin A, urolithin B, pyrocatechol, pyrogallol, Catechin, 3-O-methylgallic acid, phloroglucinol, delphinidin-3-glucoside, delphinidin-3-galactoside (as dp-3-glc), cyanidin-3-glucoside, cyanidin-3-galactoside, petunidin-3-glucoside, petunidin-3-galactoside (as pt-3-glc), peonidin-3-glucoside, peonidin-3-galactoside, malvidin-3-glucoside, malvidin-3-galactoside, pelargonidin-3-glucoside, pelargonidin-3-galactoside (as Pg-3-glc); n.d = not detected. n/a = not applicable.
